# Effects of exercise training on left ventricular systolic and diastolic function after myocardial infarction: systematic review and meta-analysis

**DOI:** 10.3389/fcvm.2025.1526326

**Published:** 2025-03-25

**Authors:** XiaoMing Zhang, Yao Mi, Mingwang Ding, Xin Gao

**Affiliations:** ^1^ The First Clinical Medical College, Gansu University of Chinese Medicine, Lanzhou, Gansu, China; ^2^ Lanzhou University, Lanzhou, Gansu, China; ^3^ Gansu Provincial Maternal and Child Health Hospital, Lanzhou, Gansu, China; ^4^ Gansu Provincial Hospital, Lanzhou, Gansu, China

**Keywords:** exercise training, left ventricular, myocardial infarction, meta-analysis, LVEF (left ventricular ejection fraction)

## Abstract

**Objectives:**

Exercise training is a rehabilitative approach to improve cardiac function in patients with myocardial infarction. However, evidence on the effectiveness of exercise training in these patients remains limited. In this meta-analysis, we aim to evaluate the extent to which exercise training improves cardiac function in patients with myocardial infarction.

**Methods:**

We conducted a systematic search of the PubMed, Embase, Cochrane Library, and Web of Science databases to compare cardiac function in myocardial infarction patients who received exercise training combined with standard pharmacological therapy. The cardiac function indicators evaluated included: LVEF, E, E/A, LVIDd, LVIDs, NT-proBNP, E' septal, GLS, and LVMI.

**Results:**

The final analysis included 12 studies with a total of 922 patients. Compared with the standard treatment group, exercise training significantly improved LVEF (MD = 3.99, 95% CI: 1.30–6.68) and *E* (MD = 3.86, 95% CI: 1.33–6.39) in myocardial infarction patients, while showing no significant improvement in the remaining indicators.

**Systematic Review Registration:**

https://www.crd.york.ac.uk/PROSPERO/view/CRD42024571194, PROSPERO (CRD42024571194).

## Introduction

One of the main causes of death and disability in the globe is myocardial infarction (MI) (1). MI is a severe manifestation of acute coronary syndromes with important effects on cardiac structure and function, particularly on left ventricular systolic and diastolic function. Although significant progress has been made in percutaneous coronary intervention (PCI), progressive postoperative left ventricular decompensation not only affects patients' quality of life, but also increases the risk of heart failure and recurrent cardiovascular events (2, 3). Cardiac rehabilitation has immense value in the treatment of cardiovascular diseases and is recommended as a rehabilitation program for myocardial infarction patients. Cardiovascular function, quality of life, risk factor, psychological status, morbidity, and mortality are all improved by exercise training, which is also a crucial part of cardiac rehabilitation and secondary prevention in patients with cardiovascular disease (4–8).

Several studies have demonstrated that exercise training dilates peripheral arteries, improves muscle function, and increases cardiorespiratory function indices such as peak oxygen consumption (VO2peak) and anaerobic threshold of respiration (VO2AT) (9, 10). There are also a large number of studies showing that appropriate exercise training can improve the cardiac function and prognosis of patients after myocardial infarction (11, 12). However, the results of current studies on the specific effects of exercise training on LV systolic and diastolic function are inconsistent (13–17). The findings' heterogeneity may have been caused by a variety of factors, including patient characteristics, exercise modes, and various study techniques. Therefore, this study sought to provide more trustworthy evidence to support clinical practice in order to optimize the rehabilitation program for patients with myocardial infarction. It did this by conducting a systematic evaluation and meta-analysis to thoroughly assess the effects of exercise training on left ventricular systolic and diastolic function following myocardial infarction.

## Methods

### Study design

This systematic review is reported according to the Preferred Reporting Items for Systematic Reviews and Meta-Analysis Programs (PRISMAP) guidelines (18). It was registered in the International Registry of Prospective Systematic Reviews (PROSPERO) on July 2024 (registration number: CRD42024571194).

### Search strategy

We performed a comprehensive search across electronic databases (PubMed, EMBASE, and Cochrane Library, Web of science). No language restrictions were applied during the search process. To ensure comprehensive coverage of relevant literature, we utilized a combination of MeSH terms and keywords in our search methodology. The search strategy included three categories: “exercise training”, “myocardial infarction”, and “randomized controlled trials”, with the same keywords and medical subject terms (Mesh) associated with the search, including “high-intensity interval training/exercise”, “aerobic interval training/exercise”, “myocardial infarction”, “acute coronary syndrome”, “ischemic heart disease”, and “randomized controlled trial (RCT)”. These were limited to studies involving human participants, and different terms used in other countries and variations in term spelling were included in the search strategy to ensure that the search covered all potentially relevant studies on the topic.

### Inclusion and exclusion criteria

Literature screening was performed according to PICOS criteria (Population/Patient; Intervention; Comparison; Outcome; Study Design). Inclusion criteria: ≥18 years of age, regardless of gender, nationality, or race; the type of study was prospective, randomized controlled study; the experimental group used exercise training as the main intervention, which included different types of exercise (e.g., aerobic exercise, resistance training, integrated training, etc.), and the control group was a non-exercise training intervention (e.g., medication, routine care, and dietary modification); systolic function and diastolic function related by echocardiographic Detailed description of the indexes by echocardiography; clear follow-up time. Exclusion criteria: cardiomyopathy, dilated cardiomyopathy, valvular disease and other serious heart diseases; myocardial infarction with enlarged cardiac structure; patients unable to carry out exercise training; no clear description of the control group; follow-up time is not clear.

### Study selection and data extraction

According to the search strategy, literature search was performed in the selected databases, and the Endnote 20 software removed duplicates and then excluded irrelevant literature by reading the titles and abstracts. The included studies were then finalized based on the inclusion and exclusion criteria. A standardized data extraction form was used to extract the title, authors, year of publication, country in which the study was conducted, study design, and indexes reflecting systolic and diastolic function of the heart. The selection of literature, data extraction, and cross-checking were performed independently by two investigators. If there was disagreement between the two, it was resolved by mutual discussion. In the absence of reported data, attempts were made to contact the lead author of the study, and the study was excluded if data were still not available. If there were more than two follow-ups, the results from the most recent intervention were considered for selection.

### Quality assessment

The quality of the included literature was assessed independently by two investigators using the modified Jadad rating scale, and if there was disagreement between the two investigators, consensus was reached by discussion with a third investigator. The Modified Jadad Scale consists of five RCTs with scores ranging from 0–7: randomization, allocation concealment, blinding, and shedding/withdrawal. scores of 1–3 were considered to be of low quality and scores of 4–7 were considered to be of high quality (19). The quality of randomized controlled trials was assessed using the Cochrane Manual 5.1.0, which evaluates six domains of random sequence generation, allocation concealment, blinding of subjects and staff and outcome assessment, incomplete outcome data, selective reporting of outcomes, and other sources of bias, and categorizes them as low risk of bias, unclear risk of bias, or high risk of bias.

### Data analysis

Systematic evaluation and meta-analysis were performed using Review Manager (version 5.4.0). Means (M) and their respective standard deviations (SD) were extracted from each study, or medians and quartiles were converted to means and standard deviations, and 95% confidence intervals (95% CI) were expressed for each effect size. Cochran's *Q* statistic and *I*^2^ test were used to evaluate inter-study heterogeneity. Heterogeneity was considered significant when *P* < 0.05 and *I*^2^ > 50% and was estimated using a random effects model, otherwise a fixed effects approach was used. Sensitivity analysis was considered to assess the robustness and reliability of the results of each study. Subgroup analysis was used to address potential sources of heterogeneity.

## Results

### Selection process and study characteristics

**Figure 1:**
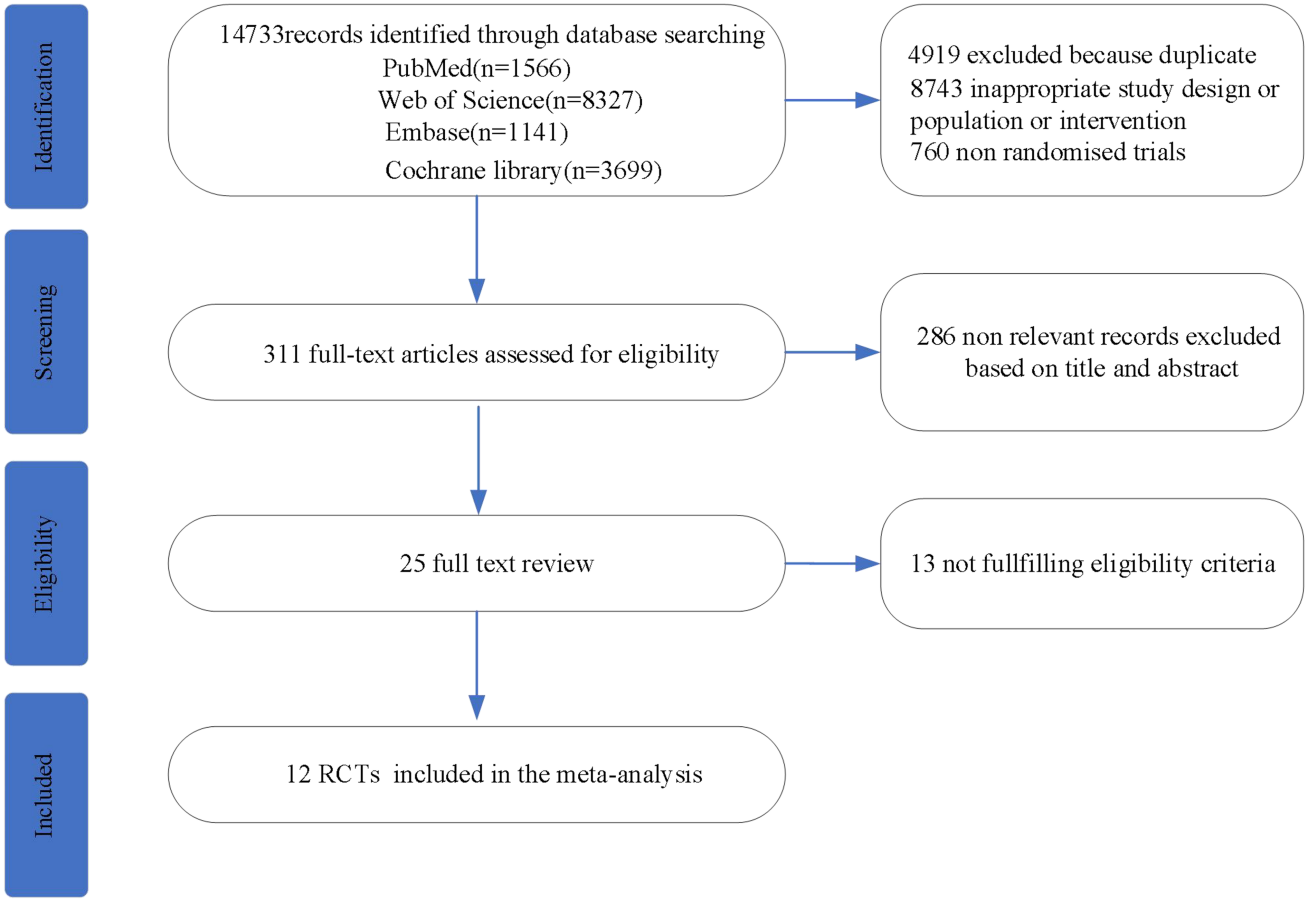
Flow chart for literature search and selection.

Literature search was performed on 4 databases (PubMed, Web of Science, Embase, Cochrane library), and a total of 14,733 articles were retrieved, 311 potentially eligible articles were retrieved after excluding duplicates and non-relevant literature, of which 286 were excluded by title and abstract, and finally 12 RCTs were included in the meta-analysis. The flowchart is shown in Figure 1.

The duration of exercise training ranged from 30 min to 90 min in the 12 transport articles included, and the studies were published between 2008 and 2022, and patient data were collected in several countries, including China, Canada, Israel, Switzerland, Portugal, and Iran. A total of 922 patients were retrieved from this study, 459 patients were randomly assigned to the exercise training group and 463 patients were assigned to the control group. The number of patients included in these studies ranged from 19–175. Other characteristics such as interventions and outcome indicators are summarized in Table 1.

**Table 1:** Characteristics of eligible studies.

Author	Year	Country	Registration number	Simple size		Age		Sex		Intervention		Training frequency	Time to start Intervention	Duration
				T	C	T	C	male	female	T	C			
C.-C. Chung	2010	China	NR	42	45	55.8 ± 12.6	59.6 ± 13.6	78	9	Exercise training	Conventional therapy	50 min, 3 sessions per week	1W	8W
P. Eser	2022	Switzerland	NCT02627586	35	34	55 (50,66)	59 (51,62)			HIIT	MICT	90 min, 3 sessions per week	4W	1Y
R.Fontes Carvalho	2015	Portugal	NCT02224495	89	86	55.4 ± 10.3	55.9 ± 10.8	144	31	Exercise training	Standard of care	70 min, 3 sessions per week	1M	8W
F. Giallauria	2008	Italy	NR	30	31	55.9 ± 3.1	55.1 ± 3.7	44	17	Exercise training	Generic instructions	40 min, 3 sessions per week	1W	6M
A. Golabchi	2012	Iranian	IRCT201011085136N1	15	14	54.20 ± 9.04	51.7 ± 6.98			Exercise training	Conventional therapy	60–90 min, 3 sessions per week	PCI-4W CABG-8W	8W
L. D. Trachsel	2019	Canada	NCT02048696	9	10	60 ± 10	57 ± 13	13	6	HIIT	Usual care	2 sessions per week		12W
M. Xiao	2021	China	NR	82	82	60.2 ± 9.2	58.7 ± 8.8	125	39	Exercise training	Conventional therapy	60 min, 3–5 sessions per week		12M
L. Xu	2016	China	NCT02584192	26	26	55.8 ± 9.7	55.5 ± 8.9	44	8	Cardiac rehabilitation	Usual care		After discharge	4W
Y. Zhang	2018	China	NR	65	65	70.3 ± 10.7	69.8 ± 10.4	113	17	Cardiac rehabilitation	Conventional drug	30–45 min, 3–5 sessions per week	2W	6M
H. Zheng	2008	China	NR	27	30					Exercise training	Routine therapy	60 min, 3 sessions per week	3–7D	6M
F. Abtahi	2017	Iranian	IRCT2016103130613N1	25	25	53.76 ± 6.96	53.6 ± 6.98	29	21	Exercise training	Usual care	60 min, 3 sessions per week	1W	8W
H. Dor-Haim	2018	Israel	NR	14	15			29		SCT	CAT	45 min		12W

**Figure 2:**
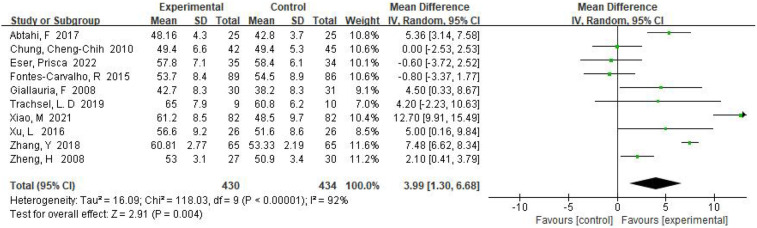
Forest plot: exercise-based cardiac rehabilitation vs. control for LVEF.

Ultimately, 10 RCTs examined the effect of exercise training on left ventricular ejection fraction (LVEF) with a total study population of 864. Based on the results of the random effects model, we found that LVEF values were significantly higher in the exercise training group compared with the control group, with an overall mean difference of MD = 3.99, 95% CI (1.30, 6.68), *I*^2^ = 92%, *P* = 0.004 (Figure 2). Analysis of the final 10 original papers revealed that the frequency of exercise training was mostly 3 times/week. The results showed that exercise training could improve left ventricular ejection fraction and myocardial contractile function in patients with myocardial infarction.

**Figure 3:**
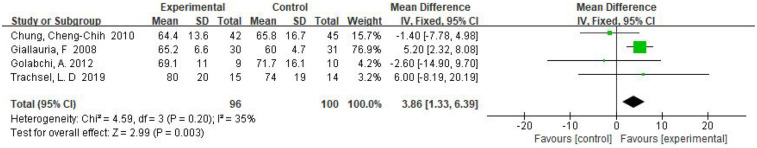
Forest plot: exercise-based cardiac rehabilitation vs. control for E.

Four RCTs examined the effect of exercise training on *E* with a total of 196 patients, and the pooled results showed that *E*-values were significantly higher in the exercise training group compared to the control group, MD = 3.86, 95% CI (1.33, 6.39) *I*^2^ = 35%, *p* = 0.003 (Figure 3).

**Figure 4:**
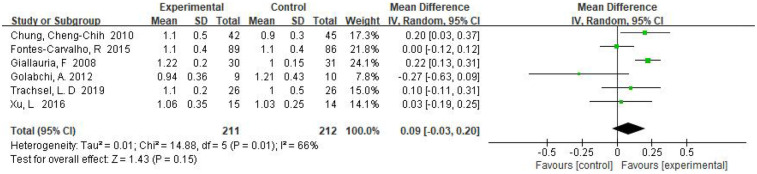
Forest plot: exercise-based cardiac rehabilitation vs. control for E/A.

Six RCTs examined the effect of exercise training on E/A with a total of 423 patients, and the pooled results showed that *E*-values were significantly higher in the exercise training group compared to the control group, MD = 0.09, 95% CI (−0.03, 0.20), *I*^2^ = 66%, *P* = 0.15 (Figure 4).

**Figure 5:**
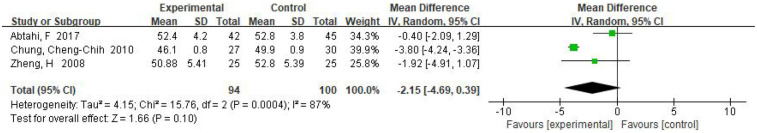
Forest plot: exercise-based cardiac rehabilitation vs. control for LVIDd.

**Figure 6:**
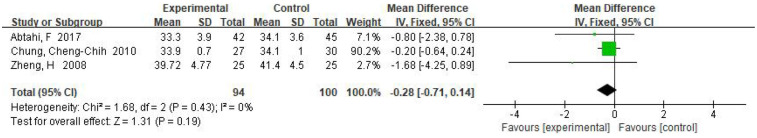
Forest plot: exercise-based cardiac rehabilitation vs. control for LVIDs.

Three RCTs examined the effect of exercise training on left ventricular end-diastolic diameter (LVIDd) with a total of 194 patients, and the pooled analysis showed that exercise training had a trend to reduce LVIDd compared to the control group, but statistically insignificant, with an overall mean difference of MD = −2.15, 95% CI (−4.69, 0.39), *I*^2^ = 87%, *P* = 0.10 (Figure 5). At the same time, exercise training did not reduce left ventricular end-systolic diameter (LVIDs), with an overall mean difference of MD = −0.28, 95% CI (−0.71, 0.14), *I*^2^ = 0%, *P* = 0.19 (Figure 6).

**Figure 7:**
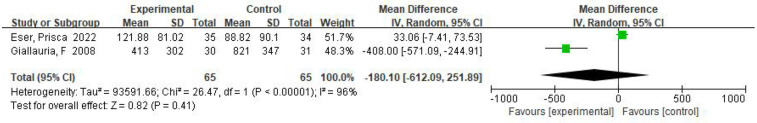
Forest plot: exercise-based cardiac rehabilitation vs. control for NT-proBNP.

Two RCTs examined the effect of exercise training on N-terminal pro-B-type natriuretic peptide (NT-proBNP) with a total of 130 patients, and the pooled results showed a trend toward lower NT-proBNP with exercise training compared with the control group, but statistically insignificant, with an overall mean effect of MD = −180.10, 95% (−612.09, 251.89), *I*^2^ = 96%, *P* = 0.41 (Figure 7).

**Figure 8:**
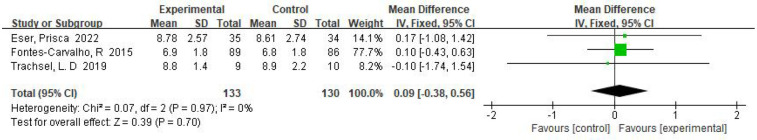
Forest plot: exercise-based cardiac rehabilitation vs. control for E′ interval.

Three RCTs examined the effect of exercise training on E′ interval with a total of 263 patients, which showed that the exercise training group did not significantly improve the E′ interval compared to the control group, with an overall mean effect MD = 0.09, 95% (−0.38, 0.56), *I*^2^ = 0%, *P* = 0.70 (Figure 8).

**Figure 9:**
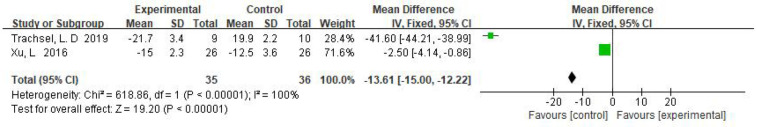
Forest plot: exercise-based cardiac rehabilitation vs. control for GLS.

Two RCTs examined the effect of exercise training on global longitudinal strain (GLS) with a total of 71 patients were included, and the results of the pooled analysis showed that exercise training did not improve GLS, overall mean difference MD = −13.61, 95% CI (−15.00, −12.22) *I*^2^ = 100%, *p* < 0.00001 (Figure 9).

**Figure 10:**
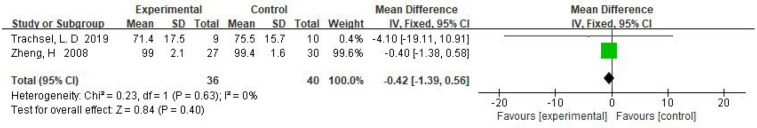
Forest plot: exercise-based cardiac rehabilitation vs. control for LVMI.

Two RCTs examined the effect of exercise training on left ventricular mass index (LVMI) with a total of 76 patients, and the pooled analysis showed a trend toward lower LVMI in the exercise training group compared with the control group, but statistically insignificant. Overall mean difference MD = −0.42, 95% CI (−1.39, 0.56), *I*^2^ = 0%, *P* = 0.40 (Figure 10).

## Discussion

So far, no treatment has been proven to improve left ventricular systolic and diastolic dysfunction after myocardial infarction (20). Exercise training enhances the protective capacity of various tissues against potential myocardial damage, such as heart infarction. Farinaz Nasirinezhad et al. found that the levels of cardiac injury markers (LDH, CKMB, Ktotal, troponin T) in patients undergoing HIIT (High-intensity interval training) were lower than those in other groups. Further research revealed that HIIT can increase cardiac protection and reduce cardiac injury by increasing the levels of G-CSF, G-CSFR, and C-kit (21). Subsequently, multiple studies have confirmed that exercise training can indeed enhance tissue protective capacity (22, 23).

Echocardiography is widely used to evaluate left ventricular systolic and diastolic function (24). Therefore, in this study, cardiac function was evaluated by echocardiography. The research by Pantaleo Giannuzzi et al. indicated that exercise can reduce left ventricular remodeling (25). Other studies have shown that exercise training can enhance the oxidative capacity of the skeletal muscle vasculature, improve endothelial dysfunction in skeletal muscle tissue in ischemic cardiomyopathy, reduce left ventricular remodeling, and help further reduce peripheral resistance and improve stroke volume (26). Recent research has demonstrated that exercise training intervenes in various stages of the inflammatory process of heart failure in myocardial infarction patients. Exercise training helps reduce the abnormal interaction between the myocardium and activated monocytes in failing patients, thus alleviating left ventricular remodeling. It also exerts a favorable control over remodeling by reducing macrophage infiltration and the expression of major circulating pro-inflammatory cytokines and their soluble receptors (27). In this study, it was found that the EF value of patients in the exercise group was significantly higher than that in the control group. The possible reason is considered to be related to the multi-dimensional synergistic effects of exercise training through mechanisms such as structural remodeling, functional enhancement, and inflammation inhibition, thereby improving LVEF.

In our study, it was found that the *E* value of the exercise training group was significantly higher compared to the control group. Previous studies believed that the early diastolic blood flow velocity *E* value at the mitral valve tip is used to evaluate left ventricular diastolic pressure. For normal people, the mitral valve inflow velocity *E* cannot be used alone to evaluate the elevation of left atrial pressure because, in the early stage of left ventricular diastole, due to the rapid decline of left ventricular pressure, the mitral valve *E* value is usually overestimated. In heart failure patients with reduced or preserved LVEF, the ratio of the early diastolic mitral valve inflow velocity (E) to the early diastolic mitral annulus velocity (e′) is the most accurate tool for evaluating left ventricular diastolic function (28). Exercise training after myocardial infarction can positively affect volume, maximum heart rate, and resting heart rate. Some research has proven that heart rate is positively correlated with velocity and negatively correlated with the *E* velocity and E/A ratio (29). Some studies have emphasized the role of heart rate as an important factor in left ventricular diastolic function. Currently, researchers generally believe that the decrease in resting heart rate caused by exercise prolongs the diastolic period, increases ventricular diastolic filling pressure, and thus increases the *E* value and E/A. In our study, it was also found that the *E* value of the exercise training group was significantly higher compared to the control group, which is consistent with the results of most previous studies. However, in this study, no improvement was found in other indicators such as E/A. The possible reason is considered to be the relatively short follow-up time, and the benefits of exercise training have not yet emerged.

Overall, the current evidence may support the improvement of left ventricular systolic and diastolic functions by exercise training after myocardial infarction. Among the included studies, 4 articles had a Jadad score of 3 points, and the remaining 8 articles had a score of 4–6 points. Therefore, the results of this study should be interpreted with caution. This study still has certain limitations. First, the types of myocardial infarction included in the 12 original studies were different. 4 articles only included STEMI, and 2 articles included both STEMI and NSTEMI patients. In the included studies, the baseline levels of patients' cardiac function indicators were different; compared with the exercise group, the treatment methods used in the control group were not the same, which may include routine care, low-intensity exercise, or no intervention at all. Among the 12 studies, the average age of the included patients ranged from a minimum of 53.76 years old to a maximum of 70.3 years old. There were 619 male patients (80.70%), which was significantly more than female patients. The time differences in the rehabilitation stage and the start time of the exercise time-window intervention were large, ranging from 3–7 days to 2 months after surgery. The follow-up time points (1-month follow-up vs. 1-year follow-up after intervention) may reflect the differences between short-term adaptation and long-term remodeling. All of the above may be potential factors affecting the differences in the improvement of left ventricular function. Second, the types and patterns of exercise are the core sources of heterogeneity. Aerobic exercises (such as walking, cycling), resistance training, HIIT, etc., have different degrees of improvement in cardiac function. The inconsistent definition of exercise intensity (such as based on percentage of maximum heart rate, VO2peak, and Borg scale) may lead to deviations in the actual intervention effect. In addition, differences in the weekly exercise frequency, single-session duration, and total exercise time may affect the cumulative effect of the results. Finally, among the studies we included, only 1 study evaluated the impact of exercise training on quality of life. Due to insufficient clinical data, other outcomes such as mental health and quality of life could not be analyzed.

## Conclusions

In conclusion, this systematic review and meta-analysis have determined that exercise training after myocardial infarction can improve left ventricular systolic and diastolic functions.

## Data Availability

The raw data supporting the conclusions of this article will be made available by the authors, without undue reservation.
